# High‐Mobility Fungus‐Triggered Biodegradable Ultraflexible Organic Transistors

**DOI:** 10.1002/advs.202105125

**Published:** 2022-03-08

**Authors:** Yahan Yang, Hongying Sun, Xiaoli Zhao, Da Xian, Xu Han, Bin Wang, Shuya Wang, Mingxin Zhang, Cong Zhang, Xiaolin Ye, Yanping Ni, Yanhong Tong, Qingxin Tang, Yichun Liu

**Affiliations:** ^1^ Center for Advanced Optoelectronic Functional Materials Research and Key Lab of UV‐Emitting Materials and Technology of Ministry of Education Northeast Normal University 5268 Renmin Street Changchun 130024 China

**Keywords:** biodegradability, crosslinking strategy, high mobility, organic transistors, ultraflexibility

## Abstract

Biodegradable organic field‐effect transistors (OFETs) have drawn tremendous attention for potential applications such as green electronic skins, degradable flexible displays, and novel implantable devices. However, it remains a huge challenge to simultaneously achieve high mobility, stable operation and controllable biodegradation of OFETs, because most of the widely used biodegradable insulating materials contain large amounts of hydrophilic groups. Herein, it is firstly proposed fungal‐degradation ultraflexible OFETs based on the crosslinked dextran (C‐dextran) as dielectric layer. The crosslinking strategy effectively eliminates polar hydrophilic groups and improves water and solvent resistance of dextran dielectric layer. The device with spin‐coated 2,7‐dioctyl[1]benzothieno[3,2‐b][1]benzothiophene (C8‐BTBT) semiconductor and C‐dextran dielectric exhibits the highest mobility up to 7.72 cm^2^ V^−1^ s^−1^, which is higher than all the reported degradable OFETs. Additionally, the device still maintains high performance regardless of in an environment humidity up to 80% or under the extreme bending radius of 0.0125 mm. After completion of their mission, the device can be controllably biodegraded by fungi without any adverse environmental effects, promoting the natural ecological cycles with the concepts of “From nature, for nature”. This work opens up a new avenue for realizing high‐performance biodegradable OFETs, and advances the process of the “green” electrical devices in practical applications.

## Graphical abstract

A high‐mobility fungus‐triggered biodegradable ultraflexible organic transistor is pioneeringly fabricated based on the crosslinked natural polysaccharide dielectrics. Profiting from this crosslinking strategy, the biodegradable organic transistors significantly boost the electrical performance and can be biodegraded controllably under the trigger of fungal decomposition. This work represents a promising prospect of crosslinked natural biomaterials for developing high‐performance and biodegradable electronic products.

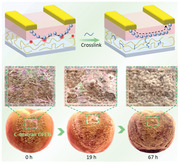

## 1 Introduction

Biodegradable organic field‐effect transistors (OFETs) whose constituents can eventually decompose into harmless byproducts, significantly alleviate environmental pollution and resource waste problems, displaying tremendous potential in new‐generation green flexible electronics.^[^ ^1^, ^2^, ^3^, ^4^ ^]^ However, the realization of the high‐mobility OFETs with a controllable degradation process is still facing an enormous challenge. Because most of the widely used biodegradable dielectric materials are polar hydrophilic functional polymers, such as poly(vinyl alcohol) (PVA),^[^ ^3^ ^]^ chitosan,^[^ ^5^, ^6^ ^]^ and cellulose.^[^ ^7^, ^8^, ^9^ ^]^ Although applying these materials as dielectric layer enables device to degrade rapidly, it is often at the cost of compromises in device performance. On the one hand, the polar hydrophilic groups of these materials are capable of trapping mobile carriers, giving rise to the inferior performance including hysteresis behavior and the deterioration of drain current.^[^ ^10^, ^11^, ^12^, ^13^ ^]^ On the other hand, the water solubility of hydrophilic groups undoubtedly leads to the invalidation of the electrical performances while the OFETs are operating in high humidity or water environments.^[^ ^14^, ^15^ ^]^ Moreover, the electric double layer (EDL) effect induced by polar groups of these dielectric materials inevitably results in the mobility overestimation at the high frequency region.^[^ ^16^, ^17^ ^]^ The abovementioned issues severely restrict the practical application of biodegradable OFETs, hindering the development of future green and sustainable electronics.

Encouragingly, the polar hydrophilic functional groups of these dielectrics can be effectively reduced by chemical crosslinking methods.^[^ ^18^, ^19^, ^20^ ^]^ Accompanying with the crosslinked process of these groups, an intricate 3D polymer network is constructed to minimize the adverse impacts of polar hydrophilic groups on device performance. Therefore, this crosslinking strategy can markedly eliminate the trap sites on the semiconductor/dielectric surface and improve the water resistance of devices, ensuring an efficient charge transport and stable operation of OFETs.^[^ ^21^, ^22^, ^23^ ^]^ For example, Ren et al. applied the a cross‐linked poly(vinyl alcohol) (C‐PVA) as the dielectric layer to successfully fabricate the high‐performance and stable OFETs, with mobility of up to 11 cm^2^ V^−1^ s^−1^.^[^ ^18^ ^]^ Unfortunately, although this crosslinking strategy of the dielectric layer can considerably improve the performance of OFETs, the consequent water and solvent resistance of dielectric materials make the device difficult to degrade by commonly reported approaches such as water or biological fluid environment degradation.

Here, based on the crosslinked natural material as the dielectric layer, we propose a fungal‐degradation OFETs which combine the advantages of high mobility and controllable biodegradation. As known to all, natural polymer materials, a kind of renewable and sustainable eco‐friendly materials derived from nature, can be readily degraded by microorganisms and treated harmlessly. By virtue of this characteristic, our crosslinked natural‐materials‐based OFETs can be completely resorbed or metabolized by widespread fungi, i.e., through safe degradation into biologically benign end products. The Nuclear Magnetic Resonance (NMR) data indicates the crosslinked devices can be biodegraded controllably under the trigger of fungal decomposition. This novel biodegradation strategy can not only enable to circumvent risks of e‐waste recycling and environmental pollution, but also promote the nutrient cycle of the natural ecosystem in accordance with the concepts of “From nature, for nature”. Through glutaraldehyde (GA) crosslinking strategy, the number of polar hydroxyl groups in the natural material is dramatically reduced, and the fabricated device exhibits excellent environmental and operational stability. Our ultraflexible OFETs can maintain field‐effect properties even under humidity conditions as high as 80%. More impressively, the elimination of hydroxyl groups also reduces interface charge carries traps, remarkably boosting the field‐effect mobility of the device to 7.72 cm^2^ V^−1^ s^−1^. This value is 15 times higher than that of uncrosslinked OFETs, and is also superior to all the reported degradable OFETs (Table S1, Supporting Information). It is conceivable that the combination of biodegradable property and high mobility would form an unprecedented OFETs system, which will be quite promising for practical application of green electronics.

## 2 Results and Discussion

### 2.1 Design of Crosslinking Strategy

**Figure 1:**
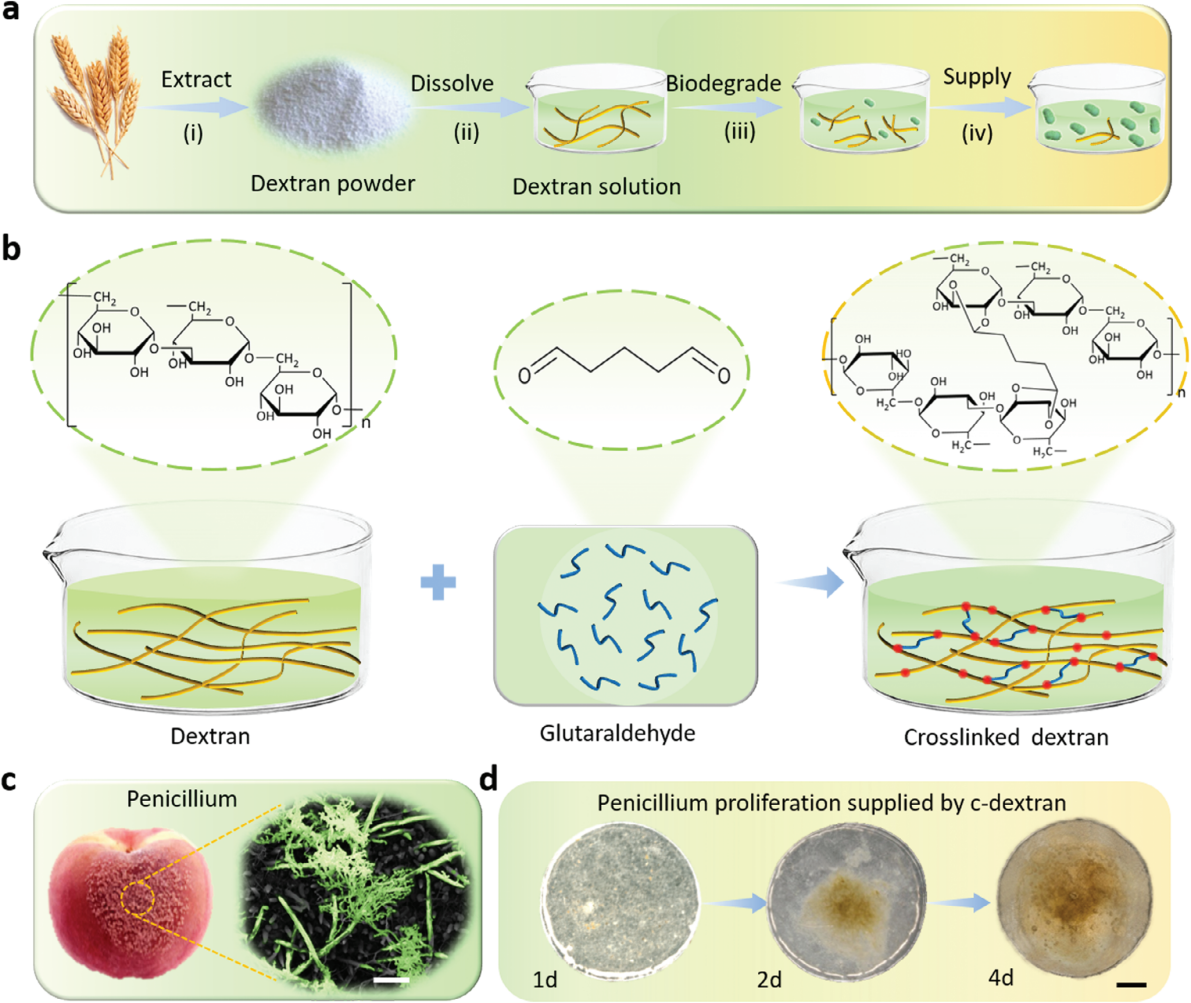
Biodegradability and renewability of dextran and its crosslinking process. a) An illustration of the sustainable life cycle of the naturally derived and biodegradable dextran. b) The mechanism diagram of the dextran crosslinking reaction, in which glutaraldehyde is introduced as a crosslinking agent. c) The picture of a rotten peach and the SEM image of Penicillium grown on the peach. Scale bar: 200 µm. d) 3D optical microscope images of Penicillium proliferation at 1, 3, and 4 days, respectively. Scale bar: 500 µm.

Dextran, a natural polysaccharide, offers remarkable advantages such as abundance in nature, biodegradability, renewability and biocompatibility.^[^ ^24^, ^25^ ^]^ In particular, natural polysaccharide as carbon source can be resorbed or metabolized by widespread fungi,^[^ ^26^ ^]^ which provides the promising perspective for the realization of the eco‐friendly electronic devices. **Figure** **1**a presents the renewable and sustainable life cycle of dextran, which can be divided into the following four processes: i) Dextran is extracted from a wide range of natural sources such as oats. ii) Dextran can be readily dissolved in water and achieve “physical disappearance” due to the existence of abundant hydroxyl groups. iii) Dextran molecules can be further decomposed by fungi. iv) The decomposed dextran molecules are absorbed and metabolized by microorganisms as nutrients eventually, which can promote the proliferation of microorganisms in nature. However, the presence of the large number of hydroxyl groups in dextran molecules seriously hinders the application of dextran as a dielectric layer in transistor devices. Therefore, we develop a crosslinking strategy to eliminate the hydroxyl groups and realize stable electrical properties and water resistance of dextran membranes. As shown in Figure 1b, the glutaraldehyde (GA) is introduced as the crosslinking agent and added into the pure dextran solution. Through the thermal treatment and thorough agitation process, an important chemical reaction, acetal reaction, should occur between the hydroxyl groups of dextran and the aldehyde groups of GA.^[^ ^27^, ^28^ ^]^ In this process, the presence of two highly reactive alpha protons makes GA more reactive and acidic in nature. The hydroxyl groups of dextran react with GA via formation of acetal bonds hence the crosslinking could take place. This crosslinking process effectively reduces the hydroxyl content of dextran, laying a foundation for the preparation of high‐performance and stable transistor devices.

Our cross‐linking strategy not only eliminates the drawbacks of the polar hydroxyl group in dextran, but also retains its biodegradable advantages. To confirm that crosslinked dextran (C‐dextran) still can be degraded and absorbed by fungi, we designed an experiment to mix C‐dextran solution with fungi, and then cultivated to explore its ability to provide nutrients for microbial proliferation. Among them, Penicillium is selected as the test sample for the fungal proliferation experiment because this fungus is widespread in nature, and commonly exists on rotten fruits, vegetables or meat.^[^ ^29^, ^30^ ^]^ Figure 1c shows an SEM image of Penicillium on a rotten peach. After cultivation under suitable conditions, we found that the Penicillium colony with C‐dextran gradually proliferated, and the whole petri dish was covered in only four days (Figure 1d). Based on this phenomenon, the quality change of penicillium colony proliferation were measured to prove this point statistically (Figure S1, Supporting Information). In contrast, the number of Penicillium in the other colony without the addition of C‐dextran solution did not change (Figure S2, Supporting Information). This phenomenon confirms that C‐dextran can be decomposed and absorbed by fungi and provides necessary nutrients for the proliferation of fungi, proving that fungal degradation is effective to C‐dextran‐based electronic products. Summarily, the whole process above represents that C‐dextran has fulfilled the mission of “From nature, to nature”, and it also demonstrates the development potential in future biodegradable and high‐performance electronic products.

### 2.2 Fabrication and Characteristics of Dextran Membranes

**Figure 2:**
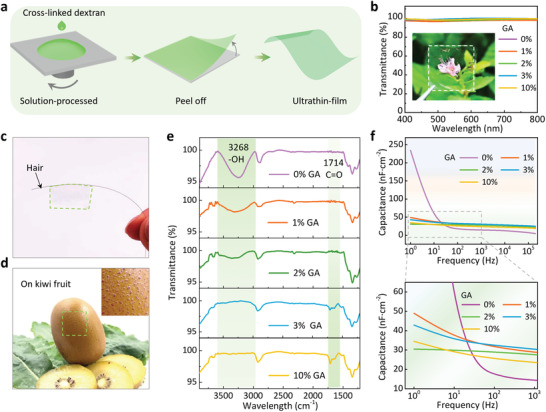
Characteristics of biodegradable dextran membranes with different GA content. a) The fabrication process of ultraflexible dextran membranes. b) The transparency of dextran membranes with different GA content in the visible spectrum. c,d) The ultralight and ultrathin dextran membrane adhered onto a hair and kiwi fruit, respectively. e) FTIR spectroscopy and f) capacitance curves of dextran membranes with different GA content.

To further demonstrate the advantages of C‐dextran, we designed a series of initial tests to compare the properties of pure dextran membranes and C‐dextran membranes with different crosslinking agent content. Based on the crosslinked dextran solution, a large‐area C‐dextran membrane was obtained via a simple spin‐coating method. Then, the membrane can be mechanically peeled off, forming a freestanding ultrathin C‐dextran membrane (**Figure** **2**a). All the resulting C‐dextran membranes (varying in GA concentration) possess high transparency of up to 96% over a visible spectrum (Figure 2b). The inset exhibits that the C‐dextran membrane is sufficiently transparent to allow a clear visualization of the plant behind the device, indicating the promising potential of our freestanding C‐dextran membrane in future transparent electronics. In addition to high transparency, our C‐dextran membrane also possesses unprecedented ultralight and conformable characteristics. The weight of the membrane is only 0.42 g cm^−2^ (Figure S3, Supporting Information), so that a single human hair can easily support a 4 cm^2^ of our ultrathin membrane with only slightly deformation (Figure 2c). To further examine the conformability on the arbitrary‐shaped objects, the C‐dextran membrane was stuck onto a kiwi fruit as shown in Figure 2d. It can be obviously observed that the membrane can seamlessly adhere to the extremely uneven surface of kiwi fruit without any bubbles and crevices. These results strongly suggest the enormous advantage of C‐dextran membranes in future implantable electronics and portable devices.

To explore the influence of different crosslinking ratios on the polar hydrophilic groups of the membranes, we carried out Fourier transform infrared spectroscopy (FTIR) of C‐dextran membranes with different GA content (Figure 2e). In Figure 2e, the large bands around 3320 cm^−1^ correspond to the ‐OH stretching of intramolecular and intermolecular hydrogen bonds of dextran.^[^ ^31^, ^32^ ^]^ Bands at 1720 cm^−1^ correspond to the backbones of ‐C = O in GA.^[^ ^33^ ^]^ It can be seen that, with the increase of GA content, the ‐OH groups absorption peak becomes weak, indeed confirming that the crosslinking reaction of the dextran/GA membranes was successfully conducted, and the content of hydroxyl peak was gradually decreasing. It is worth noting that the stretching vibration of ‐C = O becomes stronger after the GA content was higher than 2 wt%, which could be ascribed to the unreacted aldehyde group introduced by the excessive GA. In summary, when the GA content is 2 wt%, the total number of polar groups in the C‐dextran membrane is relatively minimal, which is conducive to reducing the trap density at the semiconductor/dielectric interface, achieving high charge carrier mobility in OFETs.^[^ ^34^, ^35^ ^]^

Further, the capacitance characteristics of the pure dextran membrane and C‐dextran membrane were characterized, as shown in Figure 2f. It can be observed that the frequency dependence is significantly reduced when the GA content increases, and it is almost a straight line when the GA content is 2 wt%, that is, there is no frequency dependence. However, when the GA content continues to increase, the frequency dependence of the film increases. This interesting phenomenon suggests that the EDL caused by polar hydrophilic groups is almost completely eliminated with the GA content of 2 wt%. On the contrary, other GA content will lead to the introduction of polar hydrophilic groups. The hydroxyl groups are dominant when the GA content was lower than 2 wt%, and the aldehyde groups are dominant when the GA content is higher than 2 wt%. The optimum proportion of crosslinking agent was determined to 2 wt% by FTIR tests and capacitance characteristics. All the above results confirm that the crosslinking process not only eliminates polar hydrophilic groups in the dextran membrane, but also improve the frequency dependence, showing the feasibility of further fabricating high‐performance transistor devices.

### 2.3 Solvent Resistance of C‐dextran Membranes

**Figure 3:**
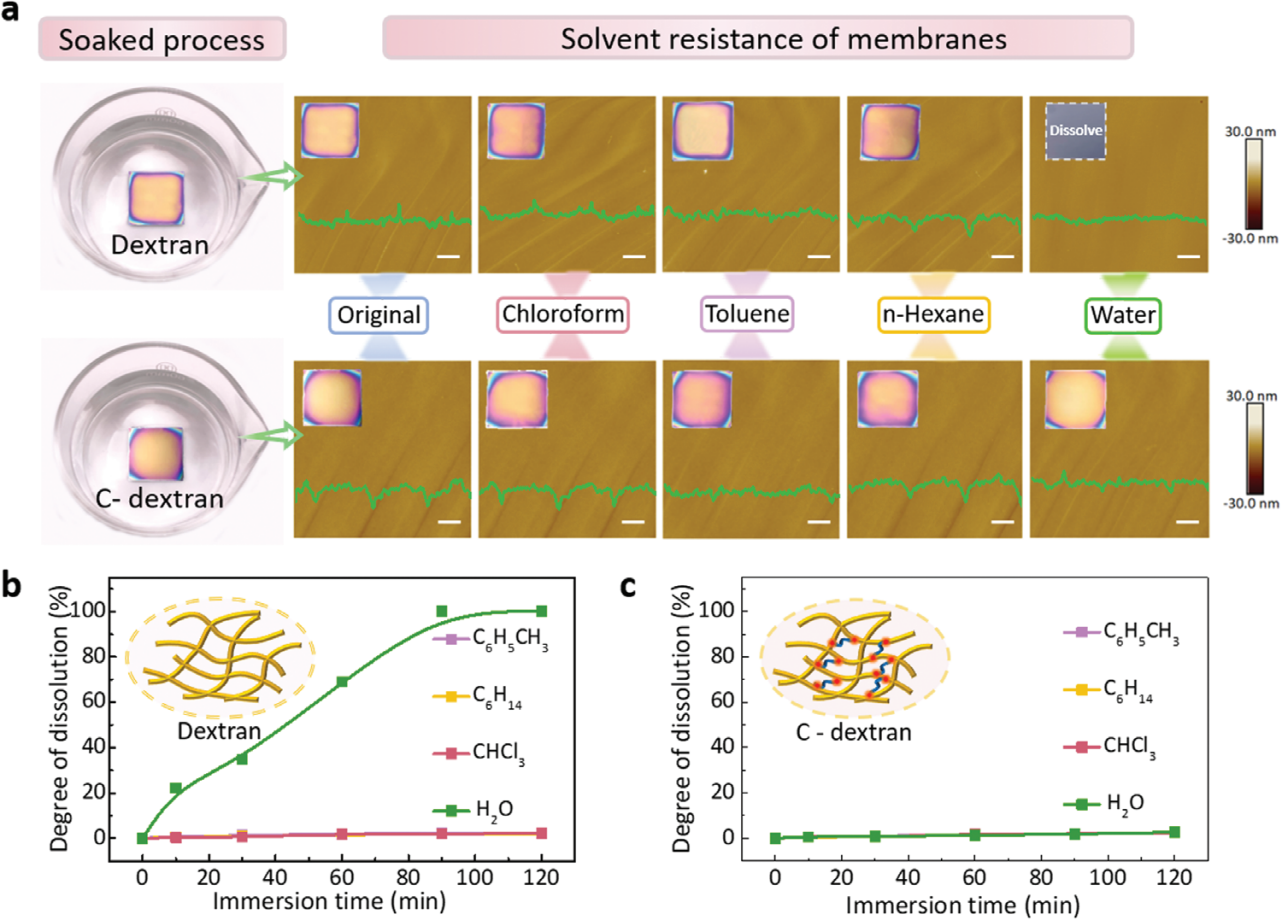
Solvent resistance of dextran and C‐dextran membranes. a) Schematic illustration of two kind of membranes immersed in different solvent respectively. Photographs, AFM images and height profiles recorded the dissolution condition of the membranes. Scale bar: 10 µm. b,c) The weight loss versus time of dextran and C‐dextran membranes in different solvent.

To indicate the environmental stability of C‐dextran membranes, the solvent resistance and water resistance tests were carried out. The concentration of GA was fixed at 2 wt%, and pure dextran membranes served as the control group. As shown in **Figure** **3**a, the dextran membrane and C‐dextran membrane were respectively immersed in different solvents such as chloroform, toluene, n‐hexane and water. After 2 h, it is observed that photographs, AFM images and height profiles of both the dextran membrane and the C‐dextran membrane remain nearly unchangeable, declaring two kinds of membranes can be stable in organic solvents without any swelling and deformation. However, when contacted with water, the pure dextran membranes immediately dissolve. Conversely, the C‐dextran membranes were immersed in water for 2 h without any changes. It suggests that incorporating with GA crosslinker in dextran can effectively improve the resistance to water. To accurately characterize the solubility of the membranes in different solvents, the weight loss of the membranes (upon soaking them in different solvent) is recorded in Figure 3b,c. The obtained membranes with different GA content were immersed in different solvent for 24 h and subsequently dried. Consistent with the results in Figure 3a, the introduction of crosslinking agent could significantly prevent the weight loss of C‐dextran membranes in water. The reason is that C‐dextran has formed an insoluble 3D polymer network after the crosslinking process, which could readily overcome the inherent hydro instability, thus making previously water‐soluble material to insoluble material.^[^ ^36^ ^]^ It can be concluded that the crosslinking strategy can effectively realize the water resistance of dextran membranes, representing enormous potential in improving the environmental stability of the biodegradable transistor device

### 2.4 Operational Stability of Dextran OFETs and C‐dextran OFETs

**Figure 4:**
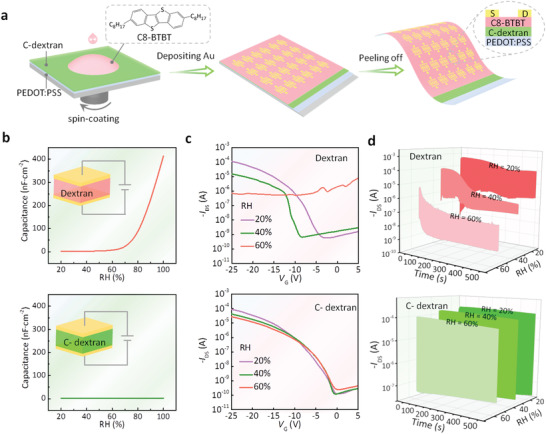
Fabrication schematic and characterization of dextran and C‐dextran transistors under different relative humidity (RH). a) Fabrication schematic of transistors with a top‐contact bottom‐gate device configuration. b) The dependence of the capacitance of the dextran and C‐dextran membranes on RH at a fixed frequency of 1 kHz. c) The corresponding transfer characteristics and d) gate voltage bias stability of dextran devices and C‐dextran devices, respectively.

The device stabilities of all types of transistors based on hydrophilic polymers remain huge challenges for scientific research and practical applications. To identify the stability of our C‐dextran OFETs, we measured the capacitance characteristics, transfer curves, and switching stability compared with the dextran‐based OFETs. The main device fabrication process is shown in **Figure** **4**a. Initially, a poly(3,4‐ethylenedioxythiophene): poly(styrenesulfonate) (PEDOT:PSS) gate electrode, C‐dextran dielectric (with the GA content of 2%), 2,7‐dioctyl[1]benzothieno[3,2‐b][1]benzothiophene (C8‐BTBT) semiconductor, and Au source/drain electrodes were sequentially deposited on the octadecyltrichlorosilane (OTS)‐modified Si substrates. Then the whole devices can be mechanically peeled off from the Si wafer, forming the ultraflexible bottom‐gate top‐contact OFET array. For the pure dextran transistors, except for the dielectric layer spin‐coated with pure dextran solution, the other fabrication process is the same as described above. Prior to the actual fabrication of the device, the capacitance characteristics of the both dielectric layers were analyzed to evaluate the insulating properties, which is essential to correctly extract the mobility value and improve the OFET field‐effect performance (Figure 4b).^[^ ^37^ ^]^ For dextran membrane, the specific capacitance increases from 2.5 to 413 nF cm^−2^ with the relative humidity (RH) increasing from 20% to 100%, indicating that the capacitance of the dextran membrane possesses a strong humidity dependence obviously. The reason for this phenomenon is that the hydroxyl group in the dextran membrane will induce the EDL effect,^[^ ^23^ ^]^ and the increasing RH further generates the larger interface capacitance. In contrast, the specific capacitance of the C‐dextran membrane will not increase with the increase of RH, and remain stable within the range of RH changes. This result is due to the consumption of the polar groups on the C‐dextran molecular chain, thereby eliminating the EDL effect at the membrane interface, so that the capacitance will not be disturbed by RH. It provides a great convenience for the accuracy of the mobility calculation of the transistor device, and avoids the drawbacks of mobility overestimation caused by environmental humidity and the invalidation of the electrical performances while the OFETs are operating in high humidity environment.

Subsequently, the transfer characteristics under different RH are compared between dextran devices and C‐dextran devices (Figure 4c). It is apparent that the dextran device continuously deteriorates as the RH increases from 20% to 40%, and almost no normal transfer characteristic is observed at 60% RH. This phenomenon indicates the significantly reduced performance. Conversely, the transfer characteristics of C‐dextran devices remained almost unchanged after the RH increasing from 20% to 60%, and only the off current increased slightly at high RH. The slight performance degradation of C‐dextran devices at high RH may be due to the instability of the organic semiconductor itself (Figure S4, Supporting Information). More importantly, the C‐dextran device can still maintain the field‐effect characteristics at the RH of up to 80%, which drastically exceed the performance of dextran devices. It robustly confirms the operational stability of the C‐dextran transistors. Furthermore, the multi‐scan transfer curves and the switching stability is also vital for assessing the device stability (Figure S5, Supporting Information and Figure 4d).^[^ ^38^ ^]^ In Figure S5 (Supporting Information), the transfer characteristic curve of the dextran device and C‐dextran devices were scanned continuously for ten times. For the dextran devices, the drain current increases continuously with the scanning numbers, and the larger hysteresis could be obviously observed. In contrast, for C‐dextran devices, the transfer curve almost completely overlapped without any drift. The switching stability is exhibited in Figure 4d. At the RH of 20%, the on/off‐state currents extracted from the transfer curves are 10^−4^ and 10^−9^, respectively, which are consistent with the initial currents in the switching characteristics. However, the off‐state current in the later period is significantly increased, which is due to the ion‐conducting behavior in the natural polysaccharide material. That is, when applying the gate voltage pulse, the mobile ions in dextran membranes are gradually accumulated to the surface of the dielectric/semiconductor layer to form an EDL. It is equivalent to the continuous electric field to induce the semiconductor channel carriers, resulting in an increasing off‐state current. Subsequently, as the humidity increases, a large amount of water molecular is adsorbed between the uncrosslinked dextran molecular chains, so that the insulating ability of the pure dextran membrane deteriorates obviously. This can be concluded from the gate leakage current in Figure S6 (Supporting Information). At RH of 40%, the gate current and drain current are of the same order of magnitude. At the RH of 60%, the gate current is two orders of magnitude larger than the drain current. Therefore, the switching characteristics at RH of 40% and 60% are not meaningful for uncrosslinked dextran devices. Especially under 60% RH, the on‐state current of the device is already positive (the on‐state current of a typical P‐type field effect transistor should be negative), because the device has lost field‐effect performance due to the severe leakage current.

However, in comparison, our C‐dextran device exhibits an outstanding stability of gate voltage bias over 400 cycles. Meanwhile, the current on/off ratio also shows a negligible shift at different RH and almost remains constant for such a long time. These results manifest that our C‐dextran devices exhibit an effective and stable field‐effect performance under different RH even up to 80% (Figure S7, Supporting Information). It suggests that, due to the elimination of environmentally‐affected polar groups, our crosslinking strategy avoids the humidity‐dependence effect in dextran devices, guaranteeing the stable operation of the C‐dextran device in the high RH range.

### 2.5 Electrical Performance Analysis of Different Crosslinking‐degree OFETs

**Figure 5:**
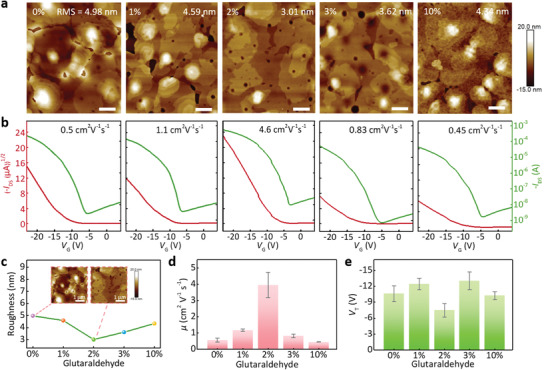
Electrical characteristics of C‐dextran transistors with different crosslinking degree. a) AFM images of organic semiconductor films (Scale bar: 1 µm) and b) corresponding transfer characteristics of transistors based on different crosslinking‐degree C‐dextran dielectrics, respectively. c) The summary roughness (Scale bar: 1 µm), d) mobility (*μ*) and e) threshold voltage (*V*_T_) versus different GA content with the device number of 50.

As known to all, the properties of the gate dielectric surface determine the semiconductor crystalline morphology and trap sites, which strongly influence the charge‐carrier transport of OFETs.^[^ ^11^, ^39^, ^40^ ^]^ Therefore, to assess the charge transport characteristics of our dextran‐based devices, we characterized the surface morphology of the C8‐BTBT on different crosslinking degrees of the dextran dielectric, and further analyzed the electrical properties of the corresponding transistors (**Figure** **5**a,b). The atomic force microscopy (AFM) images in Figure 5a present different morphologies of the C8‐BTBT films. It can be concluded that, with the increased GA content from 0 to 2 wt%, a larger grain size of the C8‐BTBT crystals was formed and a more continuous and uniform C8‐BTBT films were obtained with fewer crystal boundaries and lower root‐mean‐square (RMS) values, which is favorable for the improved mobility.^[^ ^41^, ^42^ ^]^ However, when the GA content continues to increase, the film presents an obvious discontinuity. Holes can be clearly observed between the crystal grains, resulting in more traps and hence device performance degradation at a GA content of 3 and 10 wt%. The corresponding RMS values derived from the AFM images are summarized in Figure 5c. At the GA content of 2 wt%, the RMS value of the C8‐BTBT film is only 3.01 nm, that is, the crosslinking C‐dextran dielectric is beneficial to improve the morphology and crystallization of the C8‐BTBT film. This result is not only consistent with our previous investigation of Figure 2e,f, but also in conformity to the mobility variation trend extracted from the transfer curves of devices with different crosslinking degrees (Figure 5d). To achieve more systematic and accurate comparisons, the averaged mobility and threshold voltage for both type of device with standard deviations are derived from every 10 devices and summarized in Figure 5d,e. The statistical analyzes verified the same performance trends as the above RMS values, i.e., the device based on the C‐dextran dielectric (GA content at 2 wt%) exhibited a superior electrical performance with a *μ*_FET_ of 4.6 cm^2^ V^−1^ s^−1^, maintaining a relatively low threshold voltage (*V*_T_). The increase of the mobility and the decrease of the *V*_T_ are related to the interface trap caused by the polar hydrophilic groups at dextran molecular chains. According to our previous investigation, when the content of crosslinking agent GA is 2 wt%, the content of polar groups in the dextran membrane is minimized, indicating the minimal interface traps of dielectric layers. Correspondingly, the hysteresis curves caused by interface traps can also strongly proved this point (Figure S8, Supporting Information). Therefore, the optimal C‐dextran dielectrics enable a more efficient charge transport under operating conditions, exhibiting a better electrical performance of OFETs.

### 2.6 Mechanical Robustness of Ultraflexible OFETs

**Figure 6:**
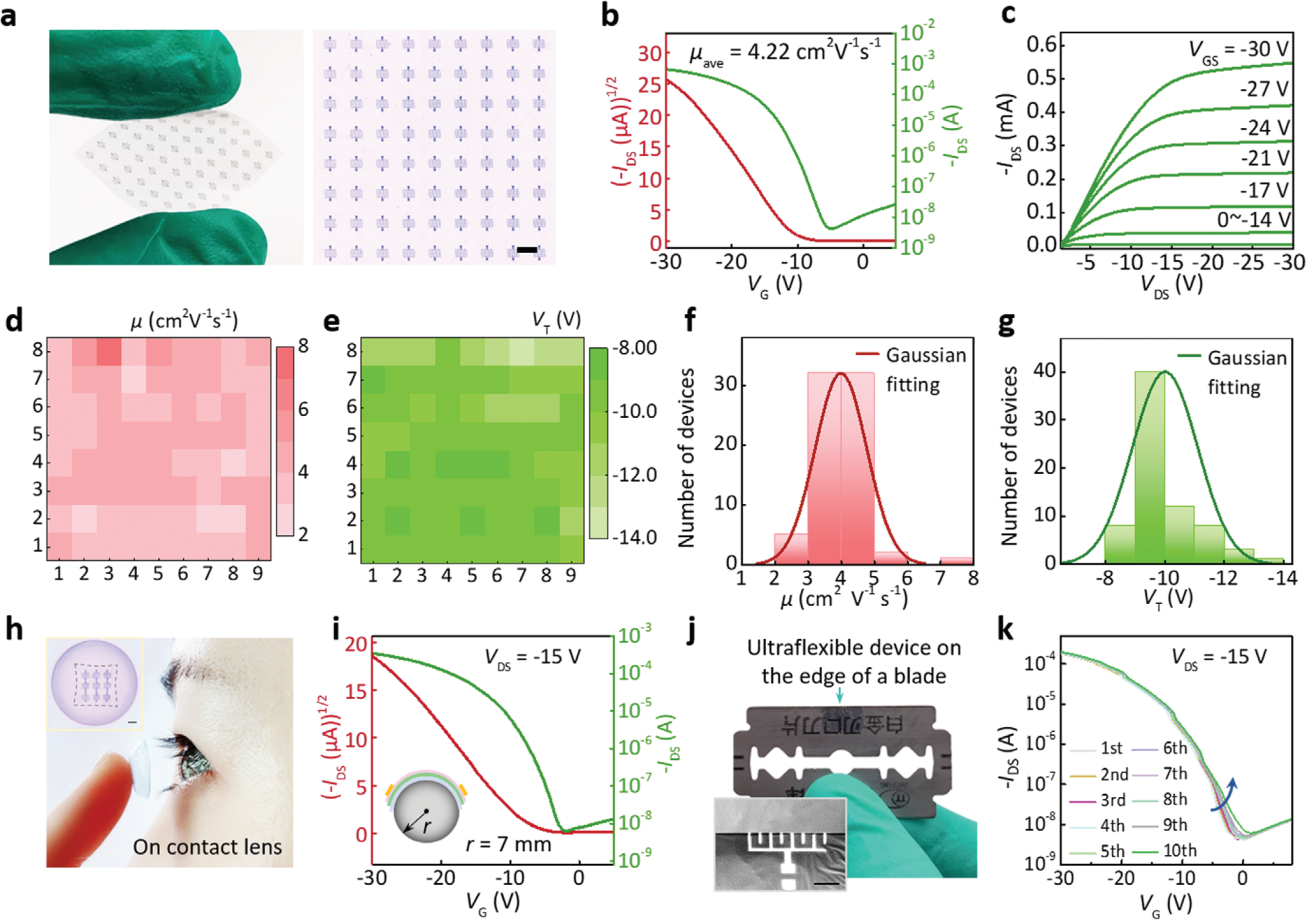
Electrical characteristics of the biodegradable OFET array. a) Photograph and the magnified transmission optical image of free‐standing OFET array (9 × 8). Scale bar: 1 mm. b,c) Typical transfer and output curves of the biodegradable OFET. d,e) Color maps of *μ* and *V*_T_ of OFET array. f,g) Histogram distributions of *μ* and *V*_T_ in 72 OFETs. h) The photograph and optical image of the biodegradable OFETs conformed onto a contact lens. Scale bar: 1 mm. i) Typical transfer curve of biodegradable OFET on contact lenses. j) Photograph of the OFETs mounted on the sharp edge of a blade. Scale bar: 500 µm. k) Multiple‐scanning transfer curves of the OFETs.

Benefiting from the excellent mechanical flexibility of dextran membranes and the preparation advantages of the spin‐coating method, we successfully utilized the C‐dextran dielectrics to fabricate the ultraflexible and large‐area OFET arrays. To verify the performance uniformity of our C‐dextran devices, we evaluated the electrical characteristics of 8 × 9 OFETs in terms of the basic transfer and output properties. The typical photograph of the OFET array and the magnified optical microscopy photograph are displayed in **Figure** **6**a. The channel length (*L*) and width (*W*) were 110 and 3150 nm, respectively. The typical transfer characteristics indicates the *p*‐type field‐effect characteristic, with the average field‐effect mobility of 4.22 cm^2^ V^−1^ s^−1^, threshold voltage (*V*_T_) of −10.43 V, and current on/off ratio (*I*_ON_/*I*_OFF_) over 10^5^ (Figure 6b). The output curve in Figure 6c presents the transistor characteristics with obvious linear and saturation current regimes. To more intuitively evaluate performance uniformity of our large‐scale devices, the spatial distributions of field‐effect mobility and threshold voltage are exhibited in Figure 6d,e, and the corresponding statistical results are summarized in Figure 6f,g, respectively. Based on these results, it could be concluded that the average saturation mobility is 4.22 cm^2^ V^−1^ s^−1^, and the highest mobility is up to 7.72 cm^2^ V^−1^ s^−1^ (Figure S9, Supporting Information). Fifty percent of 72 devices show the mobility values higher than 4 cm^2^ V^−1^ s^−1^, which meets the requirement of many practical electronic applications.^[^ ^43^ ^]^ Our C‐dextran OFET array with 100% high yield, high field‐effect characteristics, and good device performance uniformity provides an efficient way to fabricate high‐performance ultraflexible OFET array for next‐generation biodegradable electronics.

To investigate the mechanical flexibility and conformability of the C‐dextran devices, we adhered our ultraflexible devices onto a contact lens (*r* = 0.07 cm), and then tested the electrical characteristics. As shown in Figure 6h, the ultrathin thickness ensures our devices adhered on the contact lens tightly, in which the inset is the magnified 3D optical microscope image of the conformable device. The corresponding transfer curves are demonstrated in Figure 6i, indicating that even if it is subjected to a large‐magnitude bending deformation, our device can still maintain the field‐effect properties with the mobility of 1.48 cm^2^ V^−1^ s^−1^. To further exhibit the outstanding mechanical robustness of our crosslinked devices at the extreme bending state, we transferred the devices onto the sharp edge of a blade and measured the operation stability. Figure 6j displays the photograph of the ultraflexible OFETs conformed onto the sharp edge of a blade with the bending radius of 0.0125 mm (Figure S10, Supporting Information). The inset of scanning electron microscopy (SEM) confirms the tight contact between our ultraflexible device and the sharp edge of a blade. The strain of the device on the blade was calculated to be 1.14% according to the simple formula, *ε* = *d*/2*r*, where *ε* is surface strain, *d* is the thickness of devices and *r* is a bending radius. According to the transfer curves of multiple scans in Figure 6k, it can be deduced that even at the extreme bending state on the sharp edge of a blade (*r* = 0.0125 mm), the average mobility as 1.04 cm^2^ V^−1^ s^−1^ still can be obtained, and the transfer curves almost remains coincide under ten times of cycles test. These results reveal that ultraflexible C‐dextran devices can maintain their stable electrical characteristics even under extreme bending conditions, representing the great potential of high‐mobility and biodegradable OFETs in conformal intelligent systems.

### 2.7 Fungal Biodegradation Tests of the Ultraflexible C‐dextran OFETs

**Figure 7:**
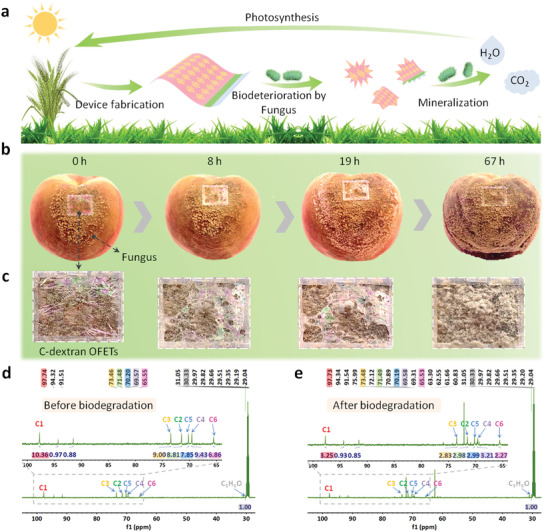
Fungal biodegradation tests of the ultraflexible C‐dextran transistors. a) The several key steps of the desired life cycle of the biodegradable natural‐materials‐based devices. b) A series photographs of C‐dextran devices on a rotten peach and c) the corresponding magnified images of the C‐dextran devices after the degradation process of 0, 8, 19 and 67 h. d,e) ^13^C‐NMR of C‐dextran solution before and after degradation, respectively.

To realize green electronics which can promote the nutrient cycle of the natural ecosystem, an ideal approach is to develop natural materials that can be derived from natural resources and eventually decomposed back to the environment.^[^ ^44^, ^45^ ^]^ **Figure** **7**a presents the several key steps of the desired life cycle of our biodegradable natural‐materials‐based devices where the dielectric layer is made from C‐dextran extracted from natural plants. Then it can be degraded via a fungal biodegradation process and sent back to the ecosphere via plants photosynthesis without any adverse environmental effects. In this work, we demonstrate the fungal biodegradation process of our C‐dextran based transistors. We attached our ultraflexible devices to rotten peaches to ensure the tight contact with the fungus, and then recorded the detailed biodegradation process in Figure 7b. After 8 h of treatment, the morphological of sample devices changes were evident. Pits and cavities appeared on the devices make our biodegradable OFETs become more fragile and thinner. Further, similar phenomena and a larger coverage area of erosion occurred after 19 h. Bigger holes with diameters of a few hundred micrometers were observed. In the end, the device has disappeared in 67 h. Figure 7c demonstrates the corresponding magnified optical photograph of the fungal degradation process which continues through formation of cavities and pits on the devices, indicating that the device has completely degraded under the action of the fungus. It is worth mentioning that, the morphology of the device attached to the unrotten peach without fungus on the surface remained unchanged (Figure S11, Supporting Information), indicating that the fungi were responsible for the device degradation. In addition, we also separately isolated Penicillium and placed it on our device to evaluate the degradation results of the device before and after exposure to the fungus (Figure S12, Supporting Information). Similarly, the eroded area of the device gradually increased until the devices were completely degraded after 9 h. To further prove the biodegradation effect of fungi on our C‐dextran devices, NMR experiments were performed on the C‐dextran before and after degradation (Figure 7d). Though the NMR spectra of dextran molecule in Figure S13 (Supporting Information), we can infer that the C‐1 to C‐6 of C‐dextran molecule are located at positions 97.74, 71.48, 73.46, 69.57, 70.20, and 65.55 ppm, respectively.^[^ ^46^ ^]^ In addition, the NMR spectra of GA and the bacterial fluid of Penicillium were also measured and exhibited in Figures S14 and S15 (Supporting Information). According to the principle of the mass balance and constant volume sampling, we compared the integral area of each carbon peak. When the C‐dextran molecules are thoroughly mixed with the Penicillium fungus liquid, the integral areas of C‐1‐C‐6 peaks are reduced by about 67%, nevertheless, the corresponding integral areas of GA peaks located at positions 94.32 and 91.51 ppm are almost unchanged. This result confirms the C‐dextran molecules have indeed been absorbed by Penicillium, indicating the promising prospect of our C‐dextran transistors in environmentally friendly applications, and provides a feasible method to explore the degradation mechanism of biodegradable devices.

**Figure 8:**
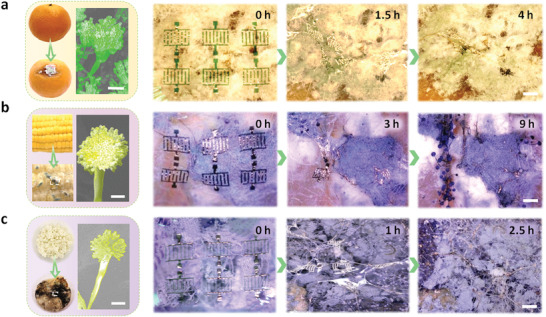
Universality of the fungal degradation for the C‐dextran devices. Photographs of the biodegradable devices attached to the moldy a) oranges, b) corn and c) cooked rice, respectively. The SEM images of Penicillium on oranges (Scale bar: 30 µm) and *Aspergillus flavus* on corn and cooked rice (Scale bar: 100 µm) are shown in left part. The time‐sequential 3D optical microscopic images in right part exhibit the biodegradable process. Scale bar: 500 µm.

With this unique degradation mechanism, a wide collection of fungi in nature are suitable for degrading our devices based on the C‐dextran dielectric. To evaluate the universality of the biodegradation of our devices, we revealed the decomposition process of our devices on moldy fruits, crops and cooked foods (represented by oranges, corn and rice, respectively). **Figure** **8**a–c exhibit the pictures of our C‐dextran ultraflexible devices when they are attached to the moldy oranges, corn and rice, respectively. In addition, the time‐sequential biodegradation images of our ultraflexible devices are subsequently recorded. With the assistance of SEM images, we respectively inferred that the fungus on oranges is Penicillium,^[^ ^47^ ^]^ and the fungus on corn and rice is *Aspergillus flavus* (Figure S16, Supporting Information).^[^ ^48^ ^]^ Figure 8a was used as an example to analyze the biodegradation kinetics of our C‐dextran devices. The optical microscopy pictures displayed the changes in the surface of the devices. Initially, the complete device was attached to on the particular area of the orange where Penicillium fungi gathered. After 1.5 h, the device was visibly eroded, and then similar phenomena and larger erosion coverage occurred. Until the 18th day of treatment, the device disappeared thoroughly, representing the complete biodegradation. The degradation behaviors of *Aspergillus flavus* in the surface of corn and rice to our devices was also carefully examined. In consistent with the erosion process as described previously, on the surface of corn and rice covered by fungi, our devices also achieved the complete degradation within a certain period of time. The unusual properties of our fungus‐triggered biodegradable devices that are opposite to operational modes in conventional electronics offer unprecedented opportunities in research areas of high‐performance eco‐friendly electronics, temporary biomedical implants, and data‐secure hardware systems.

## 3 Conclusions

In summary, a feasible strategy of C‐dextran dielectric layer is proposed for the fabrication of biodegradable ultraflexible OFETs. This crosslinking strategy significantly eliminates the hydroxyl groups of dextran polymer, improving the water resistance of dextran membranes and the electrical performance of the OFETs devices. The resultant crosslinked devices exhibit an efficient charge transport with an average mobility of 4.22 cm^2^ V^−1^ s^−1^, and the highest mobility of up to 7.72 cm^2^ V^−1^ s^−1^. Besides, the C‐dextran devices can maintain the superior stable operation at the relative humidity of up to 80%. In particular, even at the extreme bending condition on the sharp edge of a blade (*r* = 0.0125 mm), the excellent mechanical flexibility enables our biodegradable devices to maintain the average mobility as 1.04 cm^2^ V^−1^ s^−1^. Eventually, the NMR spectra certifies that the C‐dextran membranes was indeed absorbed by the fungus, that is, the whole devices can be controllably biodegraded by fungi without any adverse environmental effects. The above‐mentioned superiorities indicate that our novel strategy can fundamentally solve the challenging issues of poor performance and uncontrollable decomposition of biodegradable devices. More importantly, this strategy is expected to be extended to more natural polymer materials, such as other polysaccharides, proteins, lignin and so on, which will tremendously advance the process of the “green” electrical devices in practical applications.

## 4 Experimental Section

### Materials

Poly(3,4‐ethylenedioxythiophene): poly(styrenesulfonate) (PEDOT:PSS) aqueous solution (Clevios PH1000) was purchased from Heraeus. Ethylene glycol (EG) was obtained from Sigma‐Aldrich. Surface active agent (Capstone FS‐30) was bought from Shanghai Jianbang Industrial Co., Ltd. Octadecyltrichlorosilane (OTS, 95%) was acquired from Acros. Dextran (average Mw = 500 000 g mol^−1)^ was obtained from Shanghai Fusheng Industrial Co., Ltd. 2,7‐dioctyl[1]benzothieno[3,2‐b][1]benzothiophene (C8‐BTBT, >99%) was commercially available from Sigma‐Aldrich. Glutaraldehyde (GA, 25 wt%) was purchased from Tianjin East China Reagen.

### Device Fabrication

The Si wafers were cleaned by sonication in acetone and isopropanol for 15 min for each step beforehand, followed by drying with nitrogen. To form a self‐assembled OTS monolayer on Si wafers, the substrates treated with oxygen plasma were immersed into a OTS solution (OTS: heptane = 1: 1000 by volume) for 15 min and subsequently rinsed in pure chloroform for 1 min followed by annealing at 100 °C in a convection oven. The PEDOT:PSS solution with 6 vol% EG was spin‐coated on the OTS‐modified Si substrates at 6000 rpm for 30 s and then annealed at 100°C for 20 min. Then the PEDOT:PSS films were immersed in concentrated nitric acid for 3 min and rinsed with deionized water to serve as gate electrodes. For the fabrication of the crosslinked dextran (C‐dextran) dielectric layer, GA as the crosslinking agent was added into the dextran solution (6 wt%) according on certain proportions of 1 wt%, 2 wt%, 3 wt%, and 10 wt%, and stirred at room temperature for 10 h. The C‐dextran membrane was obtained via spin‐coating at 2500 rpm for 40 s and then annealed at 110°C for 3 h in 0.1 MPa vacuum oven. Subsequently, C8‐BTBT semiconductor was dissolved in chloroform (2 wt%) and spin‐coated at a speed of 5000 rpm for 60 s on the C‐dextran dielectric layer. Au as source and drain electrodes was deposited on the C8‐BTBT semiconductor films through shadow masks, using a thermal evaporator at a deposition rate of 0.15 Å s^−1^ 1 under 1 × 10^−5^ Torr. Eventually, the transistors can be mechanically peeled off from the OTS‐modified Si wafers with the assistance of 3M tapes, forming the ultraflexible self‐supporting transistor devices.

### Fungal Biodegradation Test

Fresh peaches were stored hermetically at the relative humidity (RH) of 50% and the storage temperature of 30 °C for seven days to obtain well‐growing Penicillium. Oranges were also stored at 50% RH and 30 °C for seven days to culture Penicillium. Corns were stored at 50% RH and 30 °C for eight days and the cooked rice were stored at 50% RH and 30 °C for four days to reproduce the *Aspergillus flavus*. Then, the ultraflexible devices are adhered on the fungal colonies and the degradation process would be recorded at different stages, respectively. For the Nuclear Magnetic Resonance (NMR) analysis, the C‐dextran membranes were vacuum dried and then dissolved in 0.55 mL of D_2_O. Among them, 100 mL Acetone was used as an internal reference. ^13^C‐NMR spectra of our sample were recorded at a base frequency of 500 MHz for analyzing the linkage composition.

### Characterization

Fourier transform infrared (FTIR) spectroscopy was collected using Nicolet 6700 by Thermo. The capacitance characteristic curves were acquired by IM 3590 chemical impedance analyzer. Optical microscopy investigations were carried out by an Olympus BX51 microscope. Scanning electron microscopy (SEM) images were obtained on a Philip XL30 instrument (Micro FEI Philips XL‐30 ESEM FEG) and postprocessed (colorized) with Adobe Photoshop CC. Atomic force microscopy (AFM) measurements were recorded with a Bruker Dimension Icon instrument (Bruker, Berlin, Germany). ^13^C‐NMR spectra were collected on an AVANCE III HD 500‐MHz spectrometer (Bruker). The transistor characteristics curves were recorded by a Keithley 4200 SCS and a Cascade M150 probe station in a shielded box at room temperature.

### Statistical Analysis

The data used for the extraction of field‐effect parameters such as *μ* and *V*_T_, was not pre‐processed before the analysis. Device outliers were excluded prior to analysis based on the reliability of the *I_S_*_D_^1/2^ versus *V*_G_ curve. All experiments had a sample size of at least *n* = 10, and statistical analysis was performed using OriginPro 9.0 (OriginLab Corporation).

## Conflict of Interest

The authors declare no conflict of interest.

## Supporting information

Supporting Information

## Data Availability

The data that support the findings of this study are available from the corresponding author upon reasonable request.
