# Supplementary material for: High‐Mobility Fungus‐Triggered Biodegradable Ultraflexible Organic Transistors
Source: Adv Sci (Weinh). 2022 Mar 8;9(13):2105125. doi: 10.1002/advs.202105125 (PMC9069197; doi:10.1002/advs.202105125)
Supplement: Supplementary file 1 — Supporting Information [file ADVS-9-2105125-s001.pdf]

ADVANCED SCIENCE
Open Access

Supporting Information

for *Adv. Sci.*, DOI 10.1002/advs.202105125

High-Mobility Fungus-Triggered Biodegradable Ultraflexible Organic Transistors

Yahan Yang, Hongying Sun, Xiaoli Zhao, Da Xian, Xu Han, Bin Wang, Shuya Wang, Mingxin Zhang, Cong Zhang, Xiaolin Ye, Yanping Ni, Yanhong Tong, Qingxin Tang* and Yichun Liu*

Supporting Information

High-Mobility Fungus-Triggered Biodegradable Ultraflexible Organic Transistors

Yahan Yang,[†] *Hongying Sun,*[†] *Xiaoli Zhao,* Da Xian, Xu Han, Bin Wang, Shuya Wang, Mingxin Zhang, Cong Zhang, Xiaolin Ye, Yanping Ni, Yanhong Tong, Qingxin Tang* and Yichun Liu*

Center for Advanced Optoelectronic Functional Materials Research, and Key Lab of UV-Emitting Materials and Technology of Ministry of Education, Northeast Normal University, 5268 Renmin Street, Changchun 130024, China.

E-mail: tangqx@nenu.edu.cn; zhaoxl326@nenu.edu.cn

Tel./fax: +86-431-85099873.

Table S1. Summary of the flexible degradable organic transistors.

Substrate	Dielectric	Semiconductor	Mobility ($cm^{-2} V^{-1} s^{-1}$)	Mechanical property	Degradation	Ref.
Natural shellac Resin	AlO_X[a]	Indigo	$1×10^{-2}$	Ultraflexible	Air	[1]
ICCNs[b]	ICCNs	C8-BTBT[c]	$7.24×10^{-2}$	Ultraflexible	-	[2]
Glass	AlO_X+BCB[d]	C_{60}	$8.8×10^{-2}$	Rigid	Thermal decomposition	[3]
PPC[e]	PPC	Pentacene	0.14	Flexible	Lipase	[4]
PVA[f]	PMTA[g]	Pentacene	0.2	Flexible	PBS buffer solution	[5]
Cellulose	Al_2O_3	PDPP-PD	0.24	Ultraflexible	PBS buffer solution	[6]
PLGA[h]	NPVA[i]	DDFTTF[j]	0.253	Flexible	Water	[7]
Paper	Parylene-C	DNTT[k]	0.33	Flexible	-	[8]
Keratin	Keratin	PCDTPT[l]	0.36	Flexible	Ammonium hydroxide	[9]
Starch paper	Parylene-C	Pentacene	0.37	Flexible	Fishbowl water	[10]
TascPLA[m]	TascPLA	DNTT	0.58	Flexible	-	[11]
Gold-coated glass slides	Almond gum	DPPTTT[n]/PMMA[o]	0.75	Rigid	-	[12]
Cellulose paper	Ion gel	P3HT[p]	0.97	Flexible	-	[13]
Levan polysaccharide and ionic liquid	Levan polysaccharide and ionic liquid	P3CPT[q]	0.98	Flexible	Deionized water	[14]
CNF[r]	PMMA	Pentacene	1.4	Ultraflexible	Fungal	[15]
Corning 7059 glass plate	Si_3N_4	A-Si:H[s]	-	Rigid	PBS buffer solution	[16]
Substrate-free	C-dextran	C8-BTBT	7.72	Ultraflexible	Fungal	Our work

[a] Aluminum oxide; [b] Ionic conductive cellulose nanopapers; [c] 2,7-dioctyl[1]benzothieno[3,2-b][1]benzothiophene; [d] CYCLOTENE 4024 resin, [e] Polypropylene carbonate; [f] Polyvinyl alcohol; [g] Poly-methacrylate tannic acid; [h] Poly(lactic-co-glycolic acid); [i] Noncrosslinked PVA; [j] 5,50-bis-(7-dodecyl-9H-fluoren-2-yl)-2,20-bithiophene; [k] Dinaphtho[2,3-b:2',3'-f] thieno[3,2-b] thiophene; [l] Poly[4-(4,4-dihexadecyl-4H-cyclopenta[1,2-b:5,4-b]dithiophen-2-yl)-alt[1,2,5]thiadiazolo[3,4-c]-pyridine]; [m] Three-arm stereocomplex polylactide; [n] Poly (3,6-di (2-thien-5-yl)-2,5-di (2-octyldodecyl)-pyrrolo [3,4-c] pyrrole-1,4-dione)thieno [3,2-b] thiophene); [o] Polymethyl methacrylate; [p] poly(3-hexylthiophene); [q] Poly[3-(5-carboxypentyl) thiophene-2,5-diyl]; [r] Cellulose nanofibrillated fiber; [s] Amorphous silicon.

S1. Quality change of Penicillium colony proliferation.

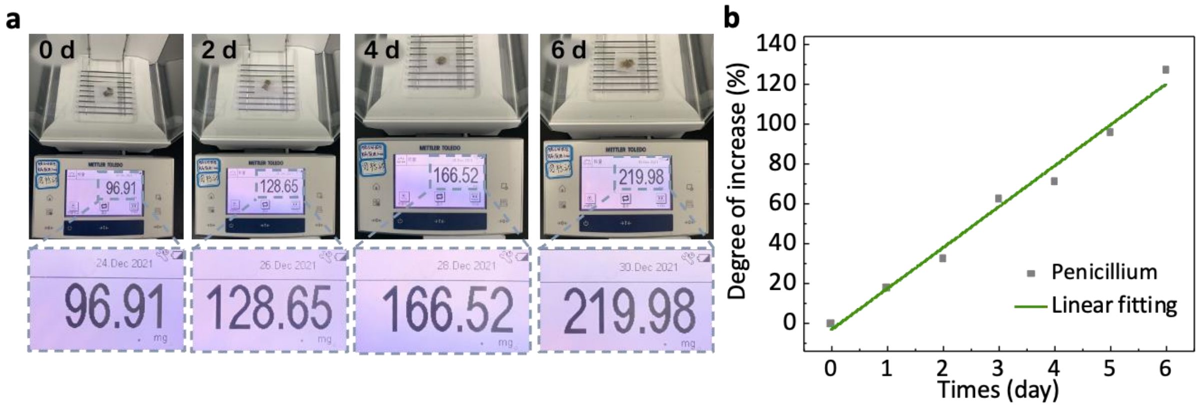

Figure S1. a) Photographs of the weighing process and b) the summary quality change of Penicillium and C-dextran cultured together for six days.

S2. Penicillium culture without C-dextran solution.

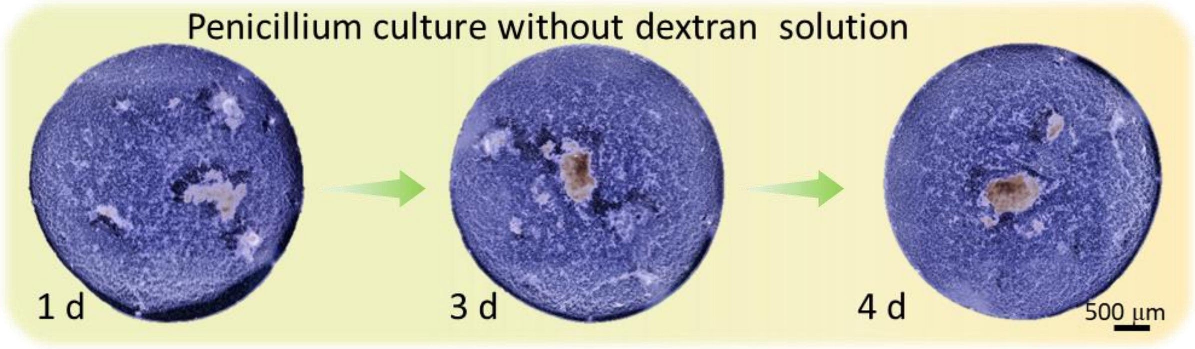

Figure S2. 3D optical images of Penicillium colony cultured without the addition of C-dextran solution. The result shows that the colony without C-dextran will not proliferate, revealing that our C-dextran as a carbohydrate is a prerequisite for fungal proliferation, that is, it can provide nutrients for fungi.

S3. Device weight measurement.

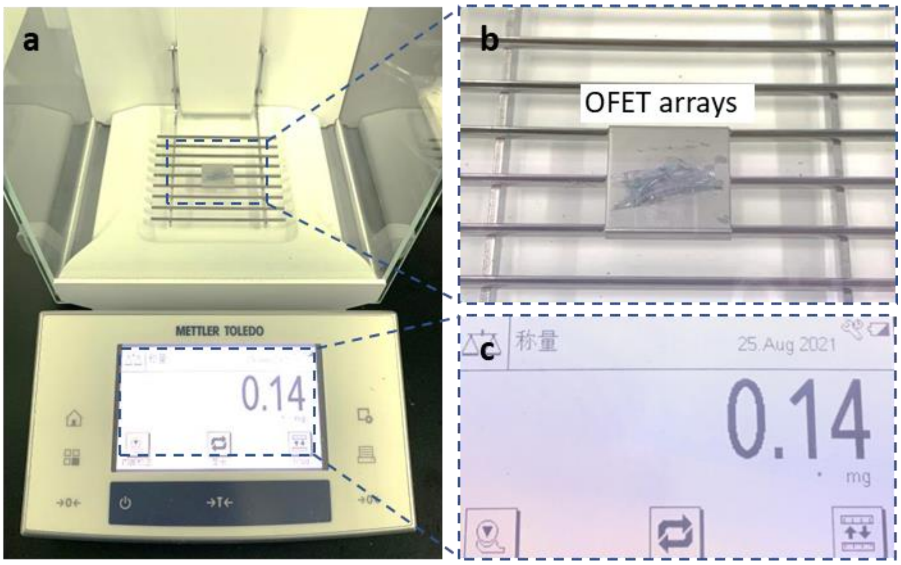

Figure S3. The weight measurement of a 2.2 × 1.5 cm^2 OFET array. The weight of the whole device array is 0.14 mg. Therefore, we can calculate that the mass of the device per unit area is 0.42 g m^{-2}.

S4. The instability of the organic semiconductor itself.

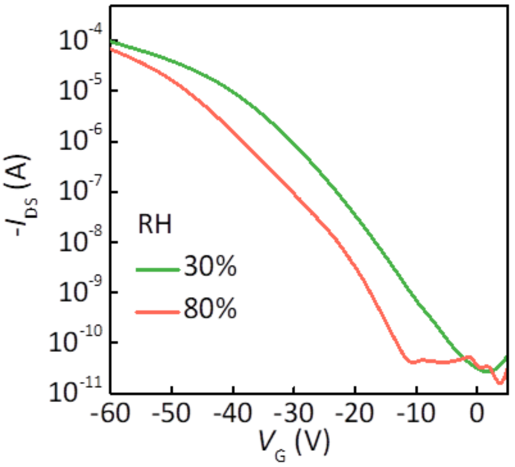

Figure S4. Transfer characteristics of a C8-BTBT OFET with OTS-modified SiO_2 as dielectric layer at different RH. It can be obviously observed that the field-effect performance of the device decreases with increasing RH, that is mainly due to the environmental instability of organic semiconductor itself.

S5. Operational stability of uncrosslinked and crosslinked devices.

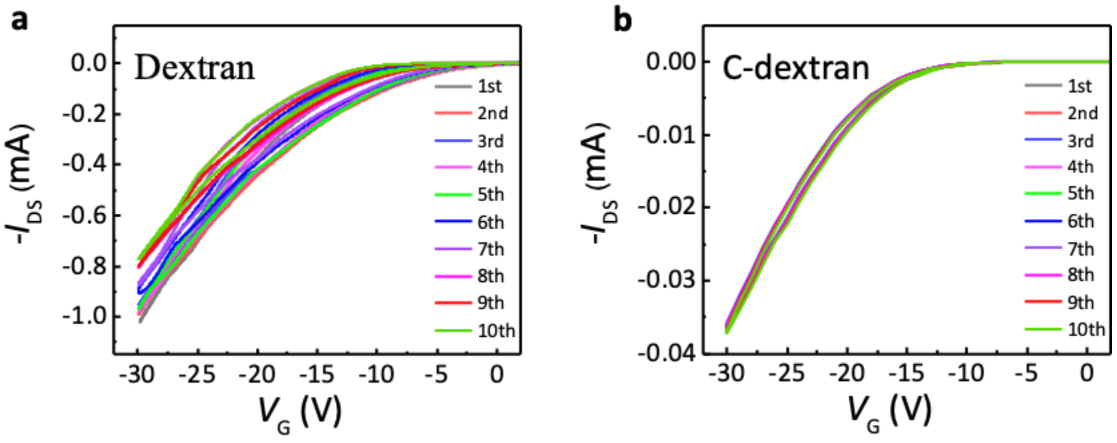

Figure S5. Multi-scan transfer curves of (a) uncrosslinked dextran devices and (b) crosslinked dextran (C-dextran) devices, respectively.

S6. Leakage current of the pure dextran device under different humidity.

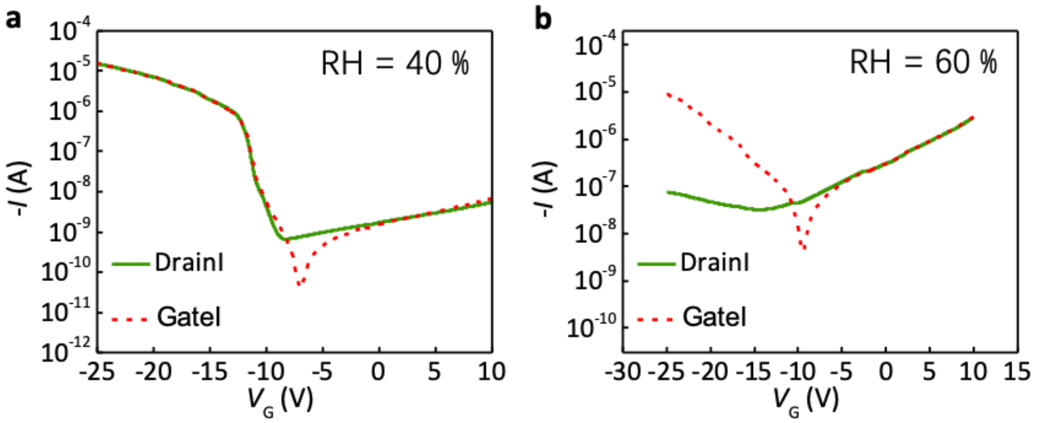

Figure S6. (a,b) Transfer curves of the pure dextran device under different RH of 40% and 60%. The dotted line represents the gate current.

S7. Operational stability of crosslinked devices under RH of 80%.

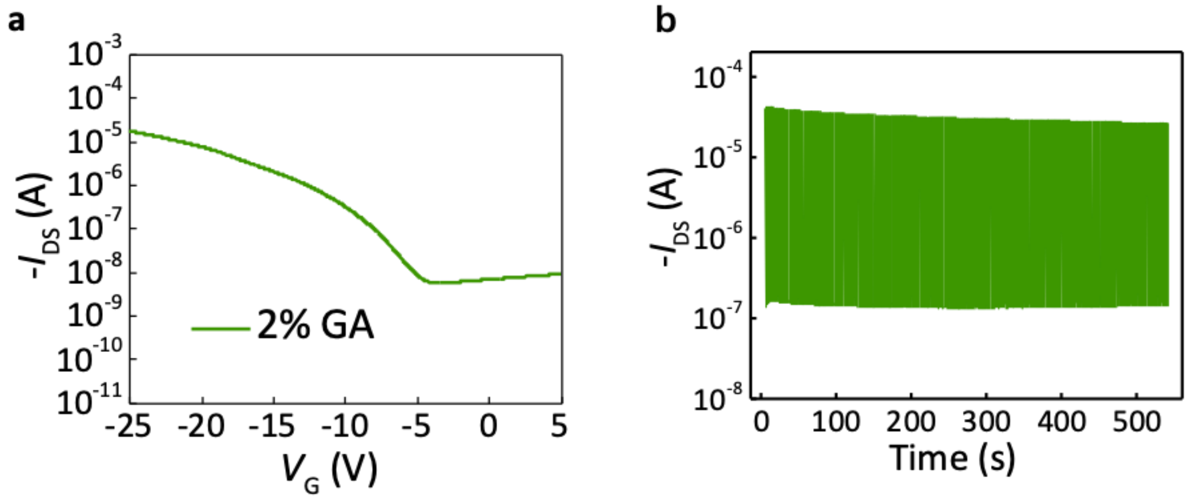

Figure S7. a) Transfer characteristics and b) gate voltage bias stability of C-dextran devices under RH of 80%.

S8. Hysteresis curves of crosslinked devices.

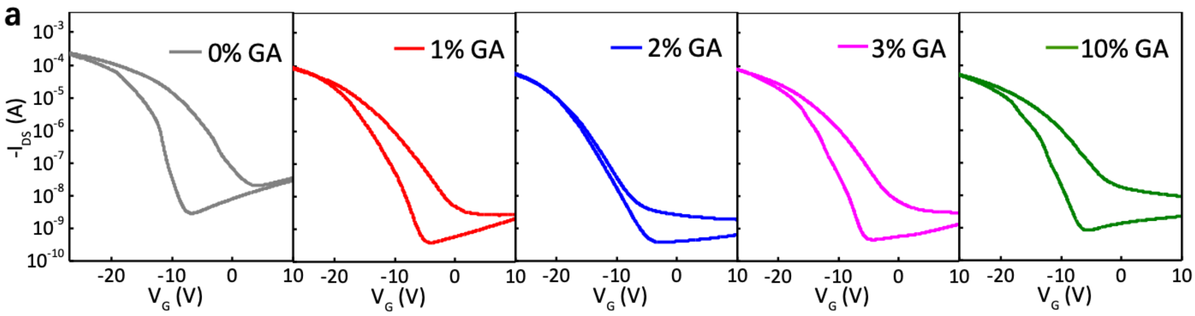

Figure S8. a) Hysteresis curves of our devices based on different crosslinking-degree C-dextran dielectrics of 0%, 1%, 2%, 3%, and 10%, respectively.

S9. The transfer curve of our transistor device.

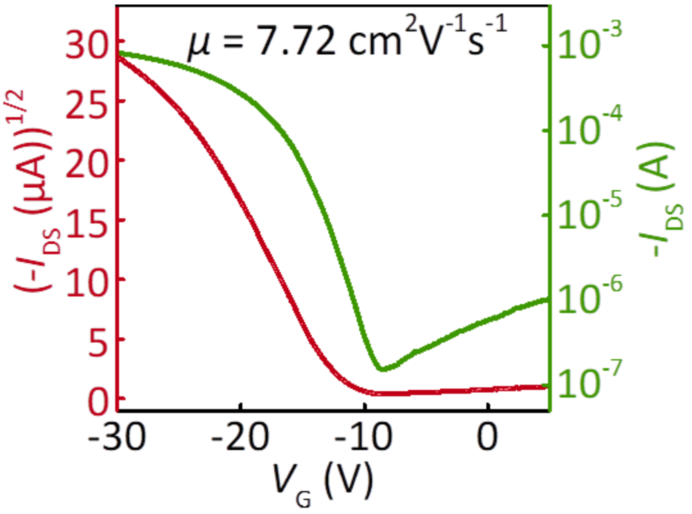

Figure S9. Transfer curves of the biodegradable OFETs. This result displays the highest mobility of our devices is up to 7.72 $cm^2\ V^{-1}\ s^{-1}$.

S10. The curvature radius of the sharp edge of a blade.

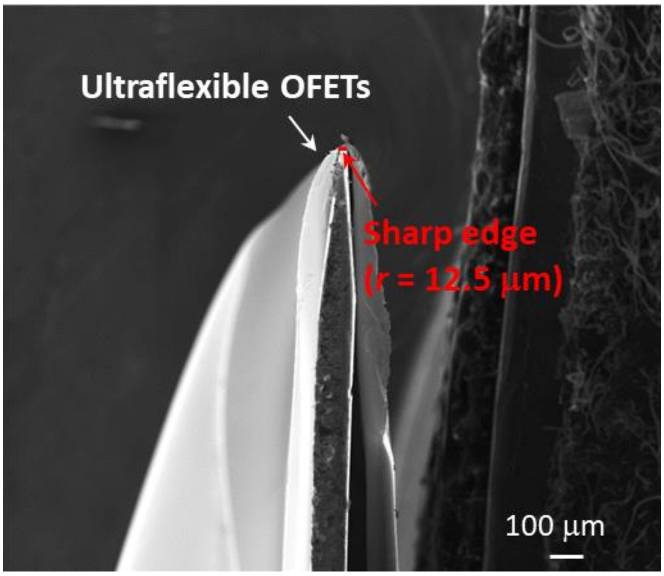

Figure S10. SEM image of OFETs mounted onto the sharp edge of a blade. The curvature radius of the sharp edge of a blade is 12.5 μm (0.0125 mm).

S11. Undegraded devices attached to the unrotten peach.

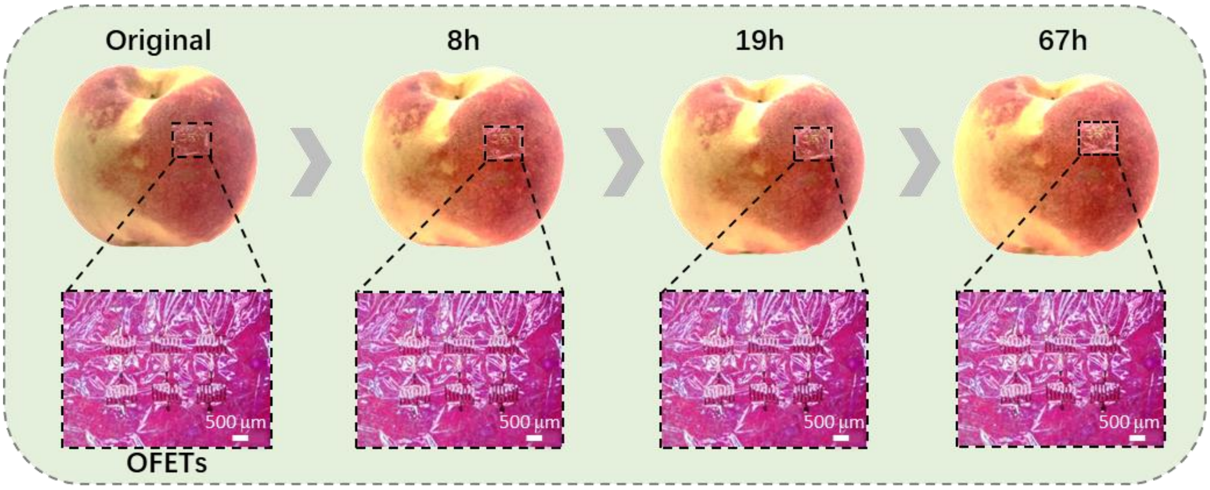

Figure S11. Photographs of the device attached to the unrotten peach. It demonstrated that the morphology of the device attached to the unrotten peach without fungi on the surface remained unchanged, indicating that the fungus was responsible for the device degradation.

S12. Degradation test before and after putting fungi on our devices.

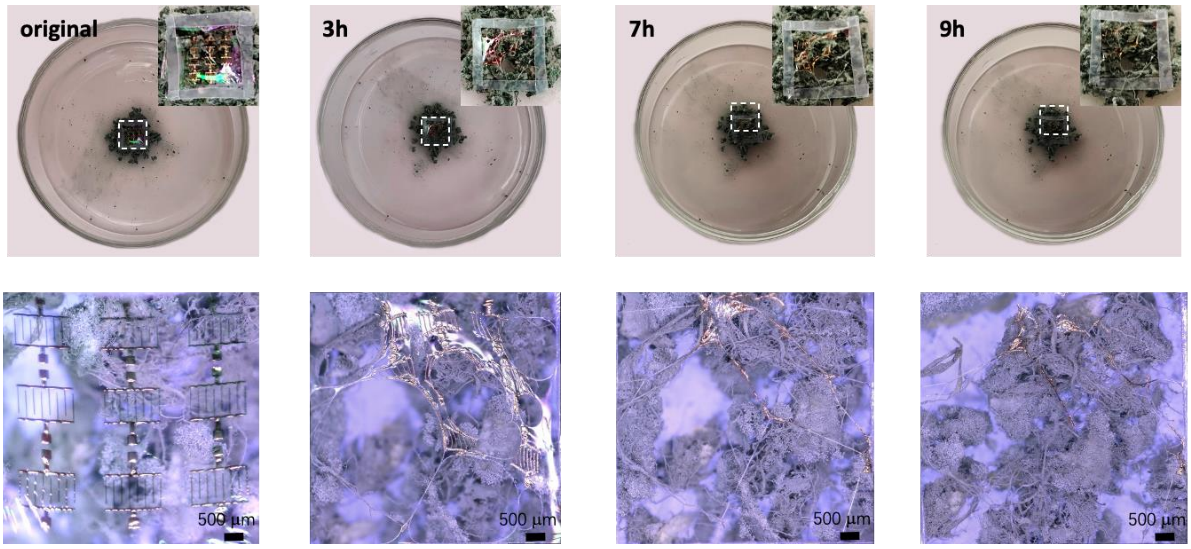

Figure S12. a) Photographs and b) 3D microscope photos of the degradation result before and after putting fungi on our device.

S13. ^{13}C-NMR of dextran solution.

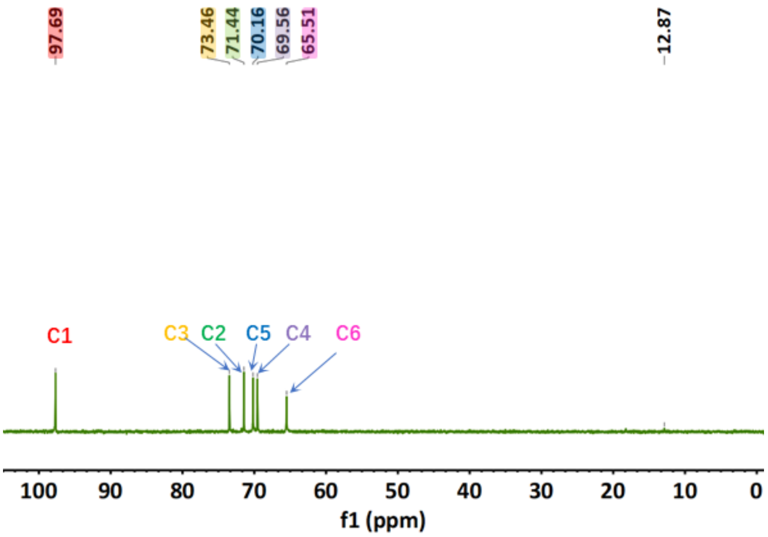

Figure S13. ^{13}C-NMR (500MHz, D_2O) spectra of dextran. The C-1 to C-6 of dextran molecule are located at positions 97.69, 71.44, 73.46, 69.56, 70.16 and 65.51 ppm, respectively.

S14. ^{13}C-NMR of glutaraldehyde solution.

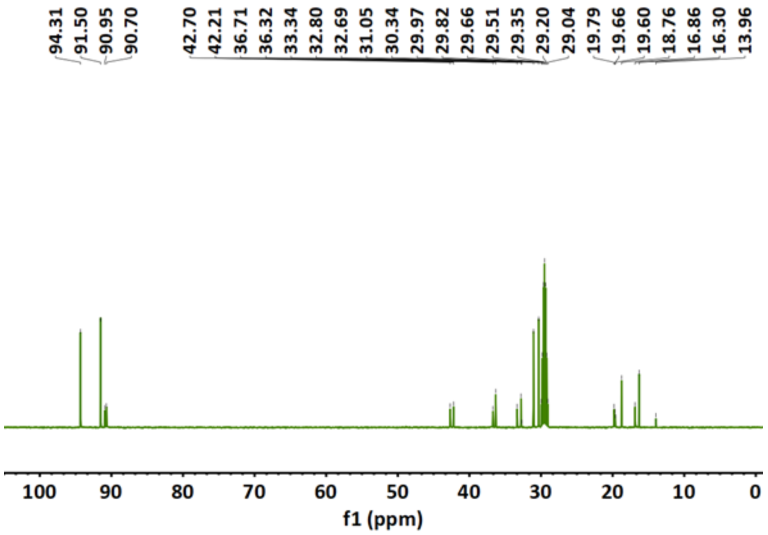

Figure S14. ^{13}C-NMR (500MHz, D_2O) spectra of glutaraldehyde. The two peaks located at positions 94.31 and 91.50 ppm are the characteristic peaks of glutaraldehyde.

S15. 13C-NMR of penicillium liquid.

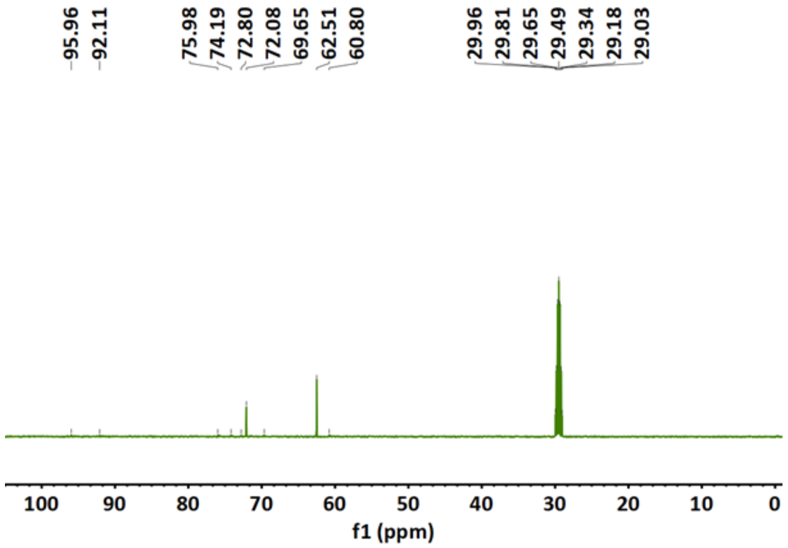

Figure S15. ^{13}C-NMR (500MHz, D_2O) spectra of penicillium liquid.

S16. The morphology of Penicillium and Aspergillus flavus.

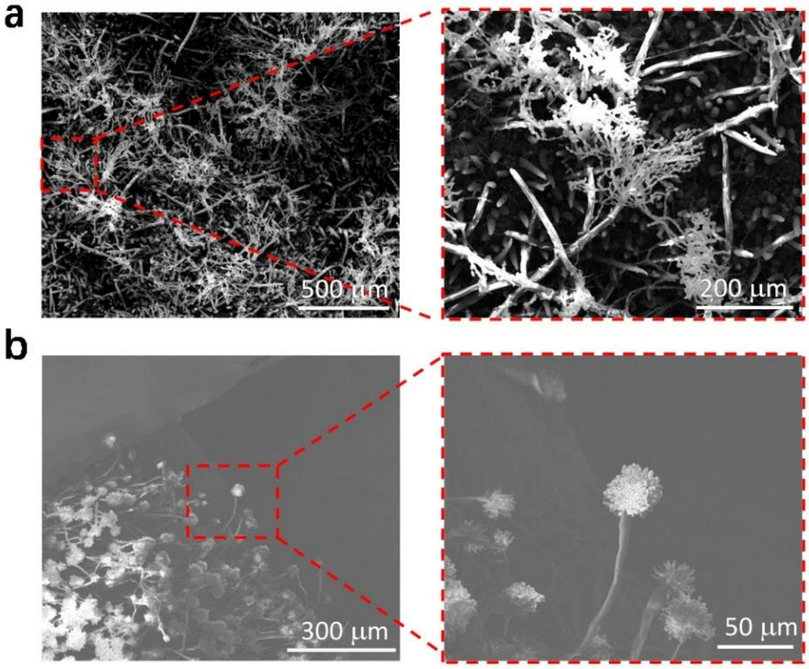

Figure S16. a) The SEM image of Penicillium and the magnified image. b) The SEM image of Aspergillus flavus and the magnified image.

Supporting Information References

[1] M. Irimia-Vladu, E. D. Głowacki, P. A. Troshin, G. Schwabegger, L. Leonat, D. K. Susarova, O. Krystal, M. Ullah, Y. Kanbur, M. A. Bodea, V. F. Razumov, H. Sitter, S. Bauer, N. S. Sariciftci, *Adv. Mater.* **2012**, *24*, 375.

[2] S. Dai, Y. Chu, D. Liu, F. Cao, X. Wu, J. Zhou, B. Zhou, Y. Chen, J. Huang, *Nat. Commun*. **2018**, *9*, 2737.

[3] A. V. Novikov, L. I. KuznetsovaI, N. N. Dremova, A. A. Parfenov, P. A. Troshin, *J. Mater. Chem. C* **2020**, *8*, 495.

[4] C. Rullyani, C.-F Sung, H.-C Lin, C.-W Chu, *Sci. Rep*. **2018**, *8*, 8146.

[5] E. Shin, J. Yoo, G. Yoo, Y.-J Kim, Y. S. Kim, *Chem. Eng. J.* **2019**, *358*, 170.

[6] T. Lei, M. Guan, J. Liu, H.-C Lin, R. Pfattner, L. Shaw, A. F. McGuire, T.-C Huang, L. Shao, K.-T Cheng, J. B.-H. Tok, Z. Bao. *PNAS*. **2017**, *114*, 5107.

[7] C. J. Bettinger, Z. Bao, *Adv. Mater*. **2010**, *22*, 651.

[8] B. Peng, X. Ren, Z. Wang, X. Wang, R. C. Roberts, P. K. L. Chan, *Sci. Rep.* **2014**, *4*, 6430.

[9] J. Ko, L. T. H. Nguyen, A. Surendran, B. Y. Tan, K. W. Ng, W. L. Leong, *ACS Appl. Mater. Interfaces.* **2017**, *9*, 43004.

[10] H. Jeong, S. Baek, S. Han, H. Jang, S. H. Kim, H. S. Lee, *Adv. Funct. Mater*. **2018**, *28*, 1704433.

[11] X. Wu, Y. Ma, G. Zhang, Y. Chu, J. Du, Y. Zhang, Z. Li, Y. Duan, Z. Fan, J. Huang, *Adv. Funct. Mater*. **2015**, *25*, 2138.

[12] M. Seck, N. Mohammadian, A. K. Diallo, S. Faraji, M. Erouel, N. Bouguila, D. Ndiaye, K. Khirouni, L. A. Majewski, *Org. Electron.* **2020**, *83*, 105735.

[13] C. Qian, J. Sun, J. Yang, Y. Gao, *RSC Adv*. **2015**, *5*, 14567.

[14] Y. J. Jo, H. Kim, J. Ok, Y.-J Shin, J. H. Shin, T. H. Kim, Y. Jung, T.-I Kim, *Adv. Funct. Mater*. **2020**, 30, 1909707.

[15] J. Park, J.-H. Seo, S.-W. Yeom, C. Yao, V. W. Yang, Z. Cai, Y. M. Jhon, B.-K. Ju, *Adv. Optical Mater*. **2018**, *6*, 1701140.

[16] Y. Tian, A. J. Flewitt, L. T. Canham, J. L. Coffer, *NPJ Mater. Degrad.* **2018**, *2*, 41.
